# No Relation between Body Temperature and Arterial Recanalization at Three Days in Patients with Acute Ischaemic Stroke

**DOI:** 10.1371/journal.pone.0140777

**Published:** 2015-10-16

**Authors:** Marjolein Geurts, H. Bart van der Worp, Alexander D. Horsch, L. Jaap Kappelle, Geert J. Biessels, Birgitta K. Velthuis, DUST investigators

**Affiliations:** 1 Department of Neurology and Neurosurgery, Brain Center Rudolf Magnus, University Medical Center Utrecht, Utrecht, The Netherlands; 2 Department of Radiology, University Medical Center Utrecht, Utrecht, The Netherlands; INSERM U894, FRANCE

## Abstract

**Background:**

Recanalization of an occluded intracranial artery is influenced by temperature-dependent enzymes, including alteplase. We assessed the relation between body temperature on admission and recanalization.

**Methods:**

We included 278 patients with acute ischaemic stroke within nine hours after symptom onset, who had an intracranial arterial occlusion on admission CT angiography, in 13 participating centres. We calculated the relation per every 0.1°Celsius increase in admission body temperature and recanalization at three days.

**Results:**

Recanalization occurred in 80% of occluded arteries. There was no relation between body temperature and recanalization at three days after adjustments for age, NIHSS score on admission and treatment with alteplase (adjusted odds ratio per 0.1°Celsius, 0.99; 95% confidence interval, 0.94–1.05; p = 0.70). Results for patients treated or not treated with alteplase were essentially the same.

**Conclusions:**

Our findings suggest that in patients with acute ischaemic stroke there is no relation between body temperature on admission and recanalization of an occluded intracranial artery three days later, irrespective of treatment with alteplase.

## Introduction

In patients with acute ischaemic stroke, recanalization of the occluded cerebral artery is strongly associated with improved functional outcome [1]. Spontaneous recanalization is influenced by temperature-dependent enzymes [2], and the *in vitro* activity of alteplase reduces with lower temperatures [3]. Whether body temperature also affects *in vivo* recanalization with or without alteplase is uncertain. This might be important, because guidelines recommend the use of antipyretics in stroke patients with fever [4, 5] and two phase III trials of therapeutic hypothermia for ischaemic stroke are in progress [6, 7]. We assessed the relation between body temperature and recanalization in patients with acute ischaemic stroke, treated with or without intravenous alteplase.

## Methods

This is a substudy of the Dutch acute Stroke study (DUST), a prospective multi-centre cohort study including adult patients with acute ischemic stroke within nine hours after symptom onset between May 2009 and July 2013. The design, eligibility criteria, and neuroimaging protocol have been reported previously [8]. All patients underwent non-contrast CT, CT perfusion, and CT angiography (CTA) within 9 hours after symptom onset. In this substudy, we included patients with visible intracranial arterial occlusion on admission CTA, and follow-up vascular imaging at 3 (±2) days. Patients who received intra-arterial treatment were excluded. Body temperature was recorded on admission.

Stroke severity at admission was assessed with the National Institutes of Health Stroke Scale (NIHSS). Stroke subtype was recorded according to the Trial of Org 10172 in Acute Stroke Treatment (TOAST) classification. Recanalization was assessed on follow-up vascular imaging and classified as no recanalization on the one hand, or partial or complete recanalization on the other. All scans were centrally evaluated by one of three experienced observers, who were blinded for the clinical data except for the side of symptoms. Poor functional outcome was defined as a modified Rankin Scale (mRS) score ≥3 at 90 days.

Temperature data were retrospectively collected from patients’ charts by one investigator, blinded for outcome measures. Body temperature on admission was defined as first recorded body temperature within twelve hours after admission, measured either tympanic or rectal.

The medical ethics committee of the University Medical Center Utrecht approved the DUST study, and written informed consent was obtained from each patient or a legal representative.

### Statistical analysis

The relation per 0.1°Celsius increase in admission body temperature and recanalization or functional outcome was calculated by means of logistic regression with a generalized estimating equations model, and expressed as an odds ratio (OR) with a corresponding 95% confidence interval (CI). We adjusted for age, NIHSS score on admission and treatment with alteplase.

Pre-defined subgroup analyses were performed with regard to treatment with alteplase and etiology of stroke. In a separate analysis patients were stratified according to the time of the second CT angiography.

Finally, we performed two additional analyses assuming that either none or all of the patients excluded because of no follow up imaging had recanalization.

## Results

Of the 1393 patients in DUST, 643 had an occluded intracranial artery on admission CTA. Reasons for exclusion for the present study were: no follow-up vascular imaging (n = 289), no admission body temperature recorded (n = 30), or intra-arterial treatment (n = 46). We included 278 patients, with 288 occluded intracranial arteries. Patient characteristics are presented in Table 1.

**Table 1:** Patient characteristics.

	Baseline characteristics n = 278
Body temperature on admission (°C)	36.7 (0.6)
Age (years)	66 (14)
Female sex	122 (44)
NIHSS score on admission	11 (7)
Previous stroke	52 (19)
Hypertension	143 (51)
Stroke etiology (TOAST)	
Large-artery atherosclerosis	118 (42)
Cardioembolism	69 (25)
Small vessel disease	0 (0)
Other	20 (7)
Unknown	71 (26)
Current smoking	90 (32)
Diabetes mellitus	28 (10)
Treatment with alteplase	187 (67)

Data are n (%), median (range), median (interquartile range (IQR)) or mean (standard deviation (SD)) where appropriate. NIHSS, National Institutes of Health Stroke Scale; TOAST, Trial of Org 10172 in Acute Stroke Treatment classification

Location of the most proximal part of the intracranial occlusion was middle cerebral artery in 215 (75%) arteries, posterior cerebral artery in 30 (10%), the intracranial part of the internal carotid artery in 18 (6%), anterior cerebral artery in 10 (4%), basilar artery in 8 (3%), and another artery in 7 (2%) patients. Ten patients had more than one occluded intracranial arteries.

Patients without follow-up imaging were older than patients with follow-up imaging (68 versus 65, P = 0.01), had higher NIHSS scores on admission (13 versus 11, P = 0.01), and had worse outcomes (median mRS 3 versus 2, P<0.001).

Follow-up imaging was performed at a median of 3 days (IQR 2 days) after admission. Follow-up imaging was CTA (95%) or MR angiography (5%). Recanalization occurred in 229 (80%) of occluded arteries. Partial or complete recanalization was associated with a better outcome compared to no recanalization (median mRS 2 (IQR 3) versus 3 (IQR2), respectively; P = 0.01).

**Fig 1:**
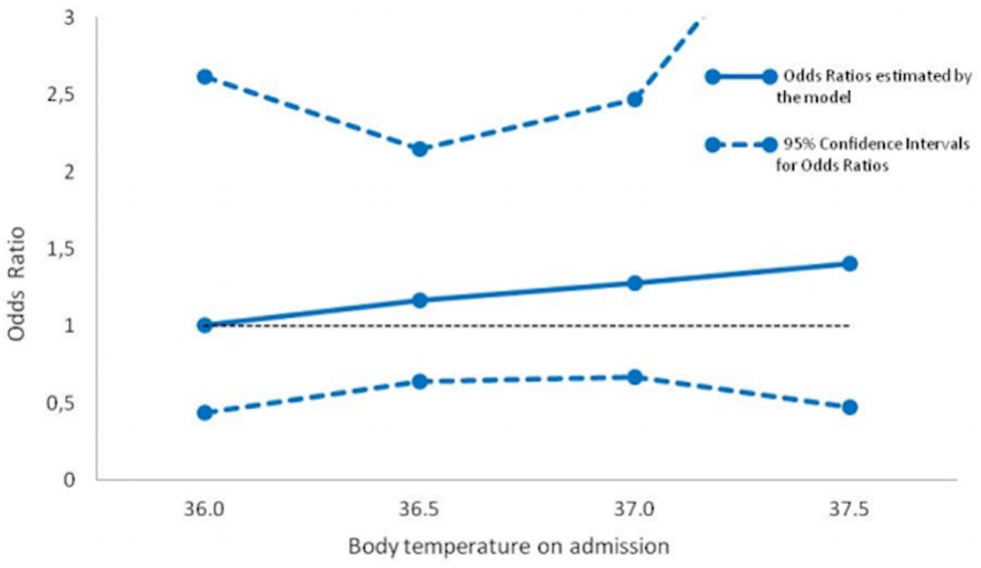
Odds ratios for the relation between body temperature on admission and recanalization.

Body temperature on admission was not associated with recanalization (OR per 0.1°C, 0.98; 95%CI, 0.93–1.03; P = 0.39; adjusted OR (aOR) per 0.1°C, 0.99; 95%CI, 0.94–1.05; P = 0.70) (Table 2, Fig 1), nor with poor outcome (OR per 0.1°C, 0.97; 95%CI, 0.93–1.01; P = 0.09).

**Table 2:** Results of unadjusted and adjusted logistic regression on the relation between body temperature and recanalization.

	Unadjusted			Adjusted for age, NIHSS, and treatment with alteplase			Test of interaction
	OR	95%CI	p	OR	95%CI	P	p
Overall analysis (n = 278)	0.98	0.93–1.03	0.39	0.99	0.94–1.05	0.70	NA
Subgroup analyses							
Treatment with alteplase (n = 187)	1.02	0.94–1.11	0.66	1.03	0.93–1.13	0.60	
No treatment with alteplase (n = 91)	0.96	0.90–1.04	0.32	0.97	0.90–1.04	0.33	0.20
Large artery atherosclerosis (n = 118)	0.94	0.87–1.01	0.10	0.95	0.87–1.03	0.22	
Cardio-embolic (n = 69)	1.01	0.90–1.13	0.91	1.02	0.88–1.19	0.76	0.42

NIHSS, National Institutes of Health Stroke Scale; OR, odds ratio; CI, confidence interval; NA, not applicable

Because follow-up imaging was missing in a substantial proportion of patients who were otherwise eligible for our study, we performed post-hoc analyses that modelled several scenarios in these patients. In these post-hoc analyses, we included all patients with a visible intracranial arterial occlusion on admission CTA, with or without follow up imaging. Patients who received intra-arterial treatment were excluded. In a best case scenario assuming that all patients without follow-up imaging had recanalization, body temperature on admission was not associated with recanalization (aOR per 0.1°C, 0.99; 95%CI, 0.95–1.04; P = 0.67). The same was found in a worst case scenario assuming that none of the patients without follow-up imaging had recanalization (aOR per 0.1°C, 1.01; 95%CI, 0.98–1.04; P = 0.50).

Results stratified by time of the second (follow-up) CT angiography are shown in S3 Table.

### Subgroup analyses

Characteristics of patients in subgroups are presented in S1 and S2 Tables. Treatment with iv alteplase was related to recanalization (aOR, 2.44; 95% CI, 1.33–4.28; P = 0.004).

There was neither difference in the relation between body temperature and recanalization between patients treated or not treated with alteplase, nor between patients with large-artery atherosclerosis and patients with cardioembolic stroke (Table 2).

## Discussion

Our findings suggest that in patients with acute ischaemic stroke, there is no relation between body temperature on admission and recanalization of an occluded intracranial artery three days later, irrespective of treatment with alteplase.

Most reports on the relation between body temperature and clot lysis concern *in vitro* studies. These have shown a reduced rate of fibrinolysis by alteplase at lower temperatures [3]. However, *in vitro* studies may not adequately reflect the *in vivo* setting of an acute arterial occlusion. Data from animal and human studies are limited. Some clinical studies found an association between higher admission body temperatures and a favourable outcome after thrombolysis with alteplase [9, 10], but others did not [11]. No studies have investigated whether this was related to higher recanalization rates.

We did not find a relation between body temperature on admission and recanalization or functional outcome. Other studies have suggested that increased body temperatures in the first few days, rather than on admission, are related to poor outcome [12, 13].

This study has limitations. Body temperature was assessed at admission and recanalization at three (± 2) days. Recanalization occurring after several hours may be of little or no benefit to ischemic tissue, and clinical consequences of delayed recanalization are therefore limited [1]. Previous studies suggest that body temperatures during the first three days may also have affected recanalization rates [14, 15]. However, in this study recanalization was strongly related to alteplase treatment within 4.5 hours, suggesting that most recanalization occurs in the first hours after stroke, and recanalization was associated with improved clinical outcome. The generalizability of our findings is hampered because in numerous patients missed follow-up imaging, but this is unlikely to have a major effect on the findings in this explanatory rather than prognostic study. We did not have data on the occurrence of infections in our population. The inter- or intra-observer variability in the measurement of recanalization was also not determined.Finally, the vast majority of our study population had normal body temperatures. Due to the limited variability in body temperatures, we could not assess associations between recanalization and body temperatures below 36.0°C or above 37.5°C.

## Supporting Information

S1 Table Baseline characteristics of patients treated and not treated with intravenous alteplase. (DOCX)

S2 Table Baseline characteristics of patients large-artery atherosclerosis and cardioembolism etiology of stroke. (DOCX)

S3 Table Results of unadjusted and adjusted logistic regression on the relation between body temperature and recanalization, stratified by time of the second CT angiography. (DOCX)
