# Supplementary material for: No Relation between Body Temperature and Arterial Recanalization at Three Days in Patients with Acute Ischaemic Stroke
Source: PLoS One. 2015 Oct 16;10(10):e0140777. doi: 10.1371/journal.pone.0140777 (PMC4608560; doi:10.1371/journal.pone.0140777)
Supplement: S1 Table — (DOCX) [file pone.0140777.s001.docx]

**S1 Table Baseline characteristics of patients treated and not treated with intravenous alteplase**

|  | Treated with iv alteplase  (n= 187) | Not treated with iv alteplase (n= 91) | P |
| --- | --- | --- | --- |
| Age (years) | 66 (14) | 67 (16) | 0.31 |
| Men | 107(57) | 49 (54) | 0.60 |
| Body temperature on admission (°C) | 36.6 (0.9) | 36.8 (0.5) | 0.06 |
| NIHSS on admission | 12 (7) | 9 (7) | 0.01 |
| Hypertension | 84 (45) | 59 (65) | 0.01 |
| Diabetes mellitus | 16 (9) | 12 (13) | 0.23 |
| Current smoking | 61 (33) | 29 (32) | 0.67 |
| Previous stroke | 24 (13) | 28 (31) | <0.001 |
| TOAST  Large-artery atherosclerosis  Cardioembolism  Small vessel disease  Other  Unknown | 83 (44)  47 (25)  0 (0)  13 (7)  44 (24) | 35 (38)  22 (24)  0 (0)  7 (8)  27 (30) | 0.69 |

Data are n (%), median (range), median (interquartile range (IQR)) or mean (standard deviation (SD)) where appropriate. NIHSS, National Institutes of Health Stroke Scale; TOAST, Trial of Org 10172 in Acute Stroke Treatment classification
