# Supplementary material for: No Relation between Body Temperature and Arterial Recanalization at Three Days in Patients with Acute Ischaemic Stroke
Source: PLoS One. 2015 Oct 16;10(10):e0140777. doi: 10.1371/journal.pone.0140777 (PMC4608560; doi:10.1371/journal.pone.0140777)
Supplement: S2 Table — (DOCX) [file pone.0140777.s002.docx]

**S2 Table Baseline characteristics of patients large-artery atherosclerosis and cardioembolism etiology of stroke**

|  | Large-artery atherosclerosis  (n= 118) | Cardioembolism (n= 69) | P |
| --- | --- | --- | --- |
| Age (years) | 67 (13) | 70 (11) | 0.17 |
| Men | 70 (59) | 44 (64) | 0.55 |
| Body temperature on admission (°C) | 36.7 (0.6) | 36.6 (1.3) | 0.63 |
| NIHSS on admission | 11 (12) | 11 (10) | 0.87 |
| Hypertension | 63 (53) | 40 (58) | 0.64 |
| Diabetes mellitus | 15 (13) | 9 (13) | 0.95 |
| Current smoking | 37 (31) | 21 (30) | 0.85 |
| Previous stroke | 24 (20) | 16 (23) | 0.65 |
| Treatment with alteplase | 83 (70) | 47 (68) | 0.75 |

Data are n (%), median (range), median (interquartile range (IQR)) or mean (standard deviation (SD)) where appropriate. NIHSS, National Institutes of Health Stroke Scale
