# Supplementary material for: No Relation between Body Temperature and Arterial Recanalization at Three Days in Patients with Acute Ischaemic Stroke
Source: PLoS One. 2015 Oct 16;10(10):e0140777. doi: 10.1371/journal.pone.0140777 (PMC4608560; doi:10.1371/journal.pone.0140777)
Supplement: S3 Table — (DOCX) [file pone.0140777.s003.docx]

**S3 Table Results of unadjusted and adjusted logistic regression on the relation between body temperature and recanalization , stratified by time of the second CT angiography**

|  | Unadjusted | | | Adjusted for age, NIHSS, and treatment with alteplase | | |
| --- | --- | --- | --- | --- | --- | --- |
|  | OR | 95%CI | p | OR | 95%CI | P |
| Day 0 (n=0) | NA |  |  | NA |  |  |
| Day 1 (n=29) | 1.00 | 0.91-1.11 | 0.96 | 1.01 | 0.91-1.12 | 0.82 |
| Day 2 (n=50) | 0.94 | 0.73-1.22 | 0.66 | 0.97 | 0.81-1.15 | 0.69 |
| Day 3 (n=114) | 0.99 | 0.91-1.06 | 0.70 | 0.99 | 0.91-1.08 | 0.72 |
| Day 4 (n=51) | 1.00 | 0.98-1.12 | 0.99 | 1.14 | 0.98-1.32 | 0.08 |
| Day 5 (n=34) | 0.91 | 0.76-1.09 | 0.30 | 0.89 | 0.72-1.10 | 0.29 |
| NIHSS, National Institutes of Health Stroke Scale; OR, odds ratio; CI, confidence interval; NA, not applicable | | | | | | |
